# Leveraging protein structural information to improve variant effect prediction

**DOI:** 10.1016/j.sbi.2025.103023

**Published:** 2025-02-22

**Authors:** Lukas Gerasimavicius, Sarah A. Teichmann, Joseph A. Marsh

**Affiliations:** 1 MRC Human Genetics Unit, Institute of Genetics and Cancer, University of Edinburgh, Edinburgh, United Kingdom; 2 Cambridge Stem Cell Institute & Dept Medicine, Jeffrey Cheah Biomedical Centre, Cambridge Biomedical Campus, University of Cambridge, Cambridge, United Kingdom; 3 Canadian Institute for Advanced Research, Toronto, Canada

## Abstract

Despite massive sequencing efforts, understanding the difference between human pathogenic and benign variants remains a challenge. Computational variant effect predictors (VEPs) have emerged as essential tools for assessing the impact of genetic variants, although their performance varies. Initially, sequence-based methods dominated the field, but recent advances, particularly in protein structure prediction technologies like AlphaFold, have led to an increased utilization of structural information by VEPs aimed at scoring human missense variants. This review highlights the progress in integrating structural information into VEPs, showcasing novel models such as AlphaMissense, PrimateAI-3D, and CPT-1 that demonstrate improved variant evaluation. Structural data offers more interpretability, especially for non-loss-of-function variants, and provides insights into complex variant interactions *in vivo*. As the field advances, utilizing biomolecular complex structures will be pivotal for future VEP development, with recent breakthroughs in protein-ligand and protein-nucleic acid complex prediction offering new avenues.

## Introduction

The vast majority of known high-risk human pathogenic mutations occur in the small percentage of the genome that contains the protein-coding regions [1]. Missense variants, which are single nucleotide changes that result in amino acid residue substitutions, directly affect the sequence, the structure, and, thus, can impact the function of the protein product and the resulting fitness of an organism [2]. However, an overwhelming proportion of missense variants appear to have little or no adverse fitness effects [3], and, despite massive sequencing efforts to capture genetic variation across diverse human populations [1,4–7], we are still failing to fully understand the factors separating pathogenic and benign variation. In fact, of the 70 million missense variants possible throughout the human genome [8], only 9.15 million have been observed [9], and even fewer, barely ~0.1%, have been annotated by clinicians as having a concrete phenotypic outcome in clinical databases [10–12]. Understanding the effects of the remaining “variants of uncertain significance” poses a fundamental challenge in genetic research and the clinic.

**Figure 1:**
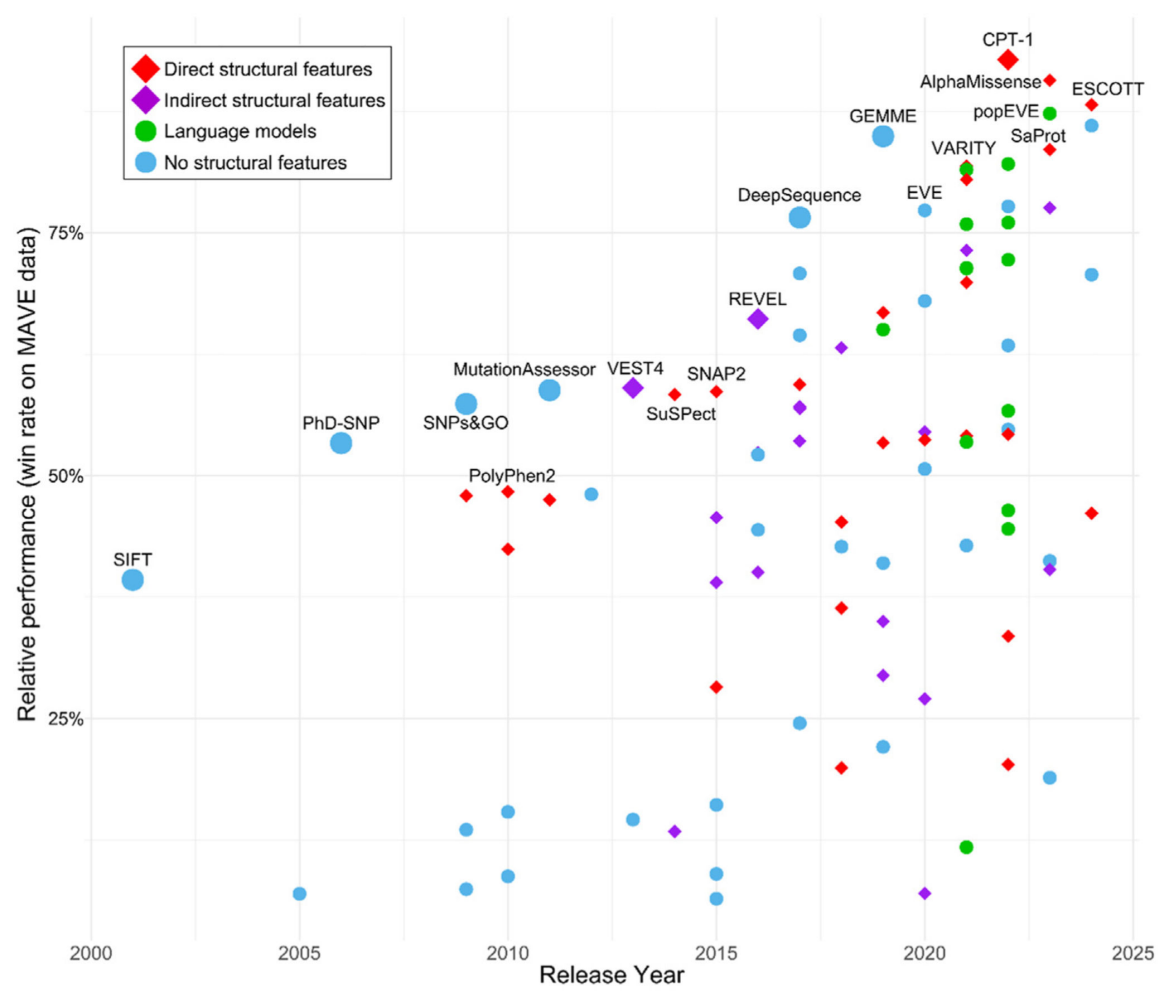
Performance of VEPs over time in comparison to the features they utilize. The performance values are represented as a percentage average win rate against other VEPs based on their correlations with experimental variant effect scores from 36 different human proteins, taken from a recent benchmarking study [14]. The release year data represents the first found mention of the method from such sources as preprint archives, code repositories, or peer-reviewed articles. Information on features used by different VEPs was compiled from the Atlas of Variant Effects Alliance VEP resource [57]. The “Indirect structural features” group are all metapredictors that use other VEPs as features, where at least one of those VEPs (usually PolyPhen-2) used structural information. “Language models” includes tools like popEVE that incorporate language models in addition to other information derived from sequence alignments, while any VEP that directly incorporated structural features was annotated as such, regardless of whether it used language models (*e.g*. CPT-1). VEPs, variant effect predictors.

While high-throughput experiments, known as multiplexed assays of variant effect (MAVEs) are being increasingly used to elucidate the effects of genetic variants [13], computational variant effect predictors (VEPs) are also widely used in the interpretation and prioritization of missense variants. Numerous VEPs have been developed, incorporating a wide variety of features. Figure 1 compares the performance relative to the year of release for 97 different VEPs, based on their correlations with MAVE data from a recent benchmarking study [14]. Importantly, this benchmark is based upon correlations with experimental MAVE data, as a means of avoiding the circularity issues that are so pervasive in the evaluation of VEP performance [15]. We emphasize that these MAVE datasets are not necessarily reflective of human pathogenicity, as what is being measured by the experimental phenotype may be different than the molecular cause of disease. Furthermore, MAVEs can cover the full variant effect landscape, whereas variance with clinically relevant effects may tend to occur at the extremes of the distribution. Despite this, the relative performance of VEPs in terms of correlations with functional assays is highly predictive of their relative performance in the identification of human pathogenic variants [14]. Importantly, similar rankings were also observed when considering only “direct” deep mutational scanning (DMS) assays (*e.g*. those that measure protein abundance or binding) or “indirect” DMS assays (*e.g*. those based on growth rate), with the same VEPs ranking highest. Moreover, the rankings show excellent agreement with another recent benchmark that used an orthogonal strategy based on the ability of VEPs to infer human traits within the UK Biobank [16].

Interestingly, over most of the history of VEP development, the top-performing methods have typically not included any structural information. While some methods that incorporated protein structural information, like PolyPhen-2 [17], SuSPect [18], and SNAP2 [19], performed relatively well in the first half of the 2010s; they were overall similar in performance to the best methods that did not include structural information. Later, methods based purely on multiple sequence alignments (MSAs), like EVmutation [20], DeepSequence [21], GEMME [22], and EVE [23], performed exceptionally well for the time. Several approaches based on large language models, either alone, such as ESM-1v [24], or in combination with sequence alignment information, such as TranceptEVE [25] and popEVE [26], have also shown strong performance in recent years, without directly incorporating any protein structural models.

Intriguingly, Figure 1 shows that, although sequence-only models dominated in terms of performance for several years, the three top VEPs from the past two years all directly include protein structural information. In this review we will highlight several factors that we believe have led to this increased performance by structure-based methods, as well as discuss some of the currently best-performing predictors leading this new generation of VEPs.

### A new generation of variant effect predictors

A major determinant of the current increase in the utilization of protein structures for variant evaluation is the current availability and ease of access of structural models for the proteome. This was made possible by the groundbreaking advances in protein structure prediction methodologies, trailblazed by AlphaFold [27,28] (AF), a convolutional neural network model using MSAs and derived through training on the Protein Data Bank to generate protein residue 3D coordinates from sequence. The method debuted at CASP13 [29] (Critical Assessment of Structure Prediction), a biannual contest that compares cutting-edge methods for predicting protein structures, outperforming the second-best method by a considerable margin. Shortly after, a redesigned AF2 [30] model was revealed at CASP14, demonstrating even further improvements with no equals, establishing it as one of the most disruptive developments in recent times [31]. Demonstrating the power beyond this framework unleashed a cascade of other structure prediction methodologies, modifying AF2 for alternative uses, or extending its limits of functionality [32–36]. Tools like RoseTTAFold [34] and AlphaFold-Multimer [35] demonstrated the capacity to accurately predict structures of protein complexes, ESMFold [37] show-cased a language model implementation for structure prediction without the need for computationally expensive MSAs, while ColabFold [32] combined and brought a number of these methodologies to the cloud.

A second factor contributing to utilization of rich structural information is the development of frameworks that transform structures or residue environments into features or embeddings that can be effectively used downstream for diverse computational biology tasks. Geometrical learning methodologies such as 3D convolutional neural nets (3DCNNs), graph neural networks (GNNs) or other specialized equivariant models are particularly suited to protein structural information, as they can directly utilize the atomistic or residue representations from structures, while directly capturing the interactions that arise from spatial contexts. More can be read about the developments in the structure-based machine learning space in a recent review by Durairaj et al. [38]. Implementations and practical examples of such frameworks are lowering the entry barrier for computational biologists, making state-of-the-art machine learning more accessible to create novel VEPs.

**Figure 2:**
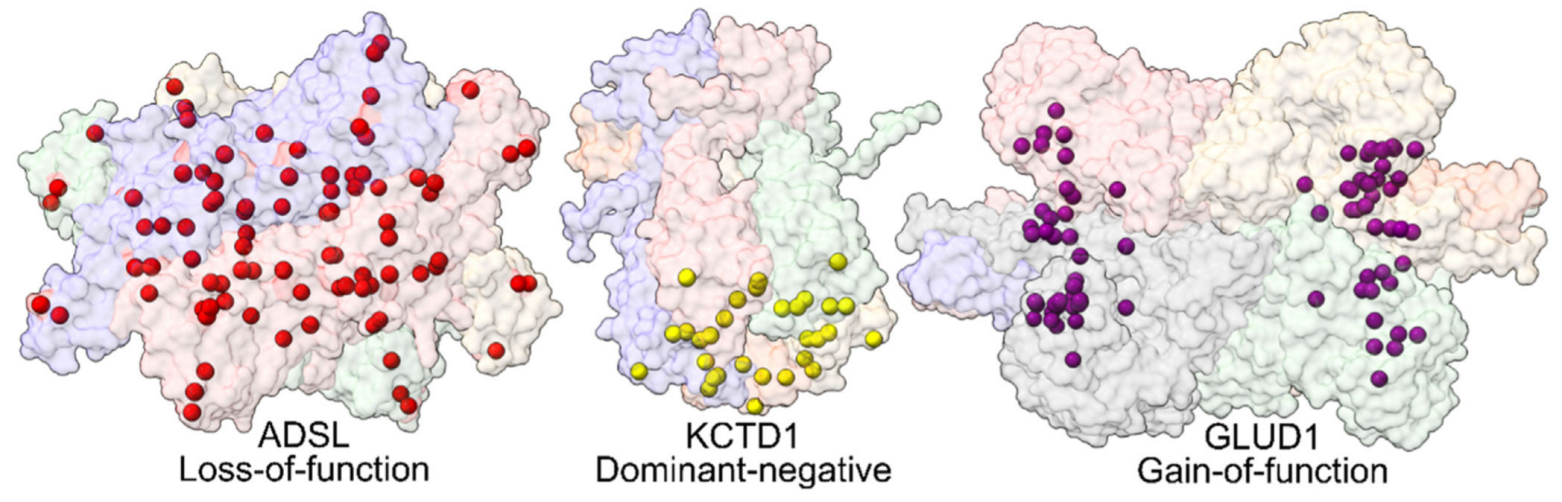
Spatial distributions of pathogenic variants in human disease genes. Three representative examples are shown here, with the sites of known pathogenic missense variants highlighted. Pathogenic variants in ADSL (PDB ID: 5nx8) are associated with an autosomal recessive disorder and act primarily via a destabilizing loss of function; thus, they tend to be broadly distributed throughout the protein structure. In contrast, missense variants in KCTD1 (PDB ID: 6s4l) and GLUD1 (PDB ID: 8sk8) act via dominant-negative and gain-of-function mechanisms, respectively, and show a high degree of spatial clustering.

Thirdly, structural information has been shown to have high-performance applications for variant effect evaluation and offers higher interpretability. While methods developed for predicting the effects of variants on protein stability tend to perform well for missense variants associated with a loss of function, nearly all VEPs underperform on gain-of-function and dominant-negative variants [39]. These can manifest disease through diverse mechanisms, like constitutive protein activation, off-target interactions, or aggregation [40]. Interestingly, such non-loss-of-function variants have been observed to show considerable clustering within protein structures (Figure 2), suggesting that utilization of this clustering may provide an avenue for improving variant effect prediction in certain genes. While most structural VEPs do not currently consider clustering, DeMAG [41] integrates both evolutionary conservation and structural proximity to known disease variant positions within its partner’s score. It adjusts the severity of the initial score for a variant based on the clinical labels in its immediate proximity, reporting improved method performance. In addition, protein secondary and quaternary structural information can be useful for predicting which human genes are most likely to be associated with gain-of-function and dominant-negative disease mutations [42].

Other works have explored loss-of-function variants to identify which of them arise due to loss of protein stability, by simultaneously comparing the signal from sequence-based evolutionary conservation tools against predictions of thermodynamic protein stability [43,44]. These approaches offer greater interpretability, as one study showed that half of the missense variants they examined caused loss of protein function by disrupting structural integrity [44]. Notably, a recent study introduced a mega-scale experiment format, termed cDNA displays proteolysis, which allowed the authors to explore the impact of an extensive number of single double missense variants on protein stability and folding within a short timeframe [45]. Such datasets provide rich information that can be utilized to more accurately model stability prediction.

Structure-based prediction has also been demonstrated to be important in cases where homologous sequence information for a target is scarce. SSEmb [46] is a hybrid VEP that integrates both sequence and structural information by combining an MSA transformer, constrained by residue spatial context and a graph neural network. The resulting methodology was shown to both outperform a sequence-only MSA transformer model as well as showing a higher degree of performance consistency across varying MSA depth.

Finally, another reason for the recent emergence of structure-based VEPs as top performers may be due to a saturation of the evolutionary signal that can be realistically further gleaned from protein sequences, at least using currently available data and methodological approaches. While the scaling laws for protein language models are continually being explored [47,48], with advances being made in model training optimization [49], alternative approaches to further increasing VEP accuracy are possible. Features that have conventionally been overlooked for the ease of sequence data, but contain additional evolutionary information and offer higher interpretability, are becoming more appealing. Despite taking unique methodological approaches, the hallmark of these recent top-performing VEPs is the utilization of at least some form of structural protein information, that grants them a performance edge over previous and even current competing predictors.

### AlphaMissense

One of the most interesting recent approaches for variant evaluation, AlphaMissense [50], combines multiple previously successful VEP strategies in a single predictor, but is also designed to take variant structural context into account, which has not been previously seen in top predictors. AlphaMissense pretraining involves a structure prediction task to train the AlphaFold network, while in parallel using the same Evoformer stack to train an unsupervised masked language model, which is the component that produces the structure-aware variant pathogenicity score. However, the resulting model is further fine-tuned for the actual variant classification task, by adapting an approach previously established in PrimateAI [51], where the training data is derived by taking a set of observed benign variation and sampling an equal amount of “pathogenic” variants from the unobserved variant pool, with replacement, and matching the trinucleotide context of the benign variant distribution. Both human and primate benign variation is pooled for the fine-tuning step, greatly enlarging the training dataset available for machine learning approaches. Finally, the variants were further weighted in the loss function by their allele frequency, with rare variant impact being reduced, a population-tuning strategy that has previously been shown to succeed in other well-performing VEPs [52].

While AlphaMissense does not directly utilize experimental or precomputed protein structures as input, the model itself learns the underlying structural context representation from sequence by being built upon a modified AlphaFold2 Evoformer framework. While AlphaFold itself was not designed for variant evaluation, as has been seen from its inability to produce mutant protein structures that would deviate from the underlying wild-type structure [53,54], it contains the building blocks to capture complex structural relationships from sequence, which can be creatively utilized for a downstream VEP task. AlphaMissense also does not produce depictions of mutant structures, but it provides a quantitative measure of predicted pathogenicity, which outperforms previous state-of-the-art methods in independent benchmarks [14].

### PrimateAI-3D

In contrast, the redesigned 3D version of PrimateAI [51] directly utilizes MSAs and structural data as input, with the authors describing the method as a semi-supervised 3D convolutional neural network for variant pathogenicity prediction [55]. The structural inputs are discretized into a grid of *2* Å voxels centered on the variant residue, with the contents of each voxel being represented in a feature vector that describes the atomic and residue conservation environment. PrimateAI-3D integrates information from the AlphaFold DB, which allows it to leverage predicted local distance difference test (pLDDT) values, a measure, which indicates the per-residue confidence of modeling and corresponds to structural order [56]. It uses features such as the shortest distance from each residue type to the voxel center, the pLDDT of the environment, amino acid frequencies for the position nearest to the voxel, combining diverse sources of information in a spatial context. The neural network architecture integrates all these features through sliding convolution operations that capture the interactions and impacts of the immediate spatial variant neighborhood. The network is trained using an analogous framework to PrimateAI, utilizing benign variants in humans and primates, and sampling unobserved human variants as a “pathogenic” set. The authors demonstrate that the structure-based model considerably outperforms or is equivalent to the ablated language model-only version, and also outperforms most predictors of the last generation, even without population tuning. However, despite its promising self-reported performance, the utility, and the broader applicability of this method are somewhat limited by its licensing terms, unlike other methods where scores are freely available without restrictions [57], thus explaining its absence from the benchmark on which Figure 1 was based. Notably, in the recent Critical Assessment of Genome Interpretation (CAGI) Annotate-All-Missense challenge, it performed well, but worse than AlphaMissense, in discriminating between pathogenic and benign variants across all allele frequency ranges [58].

### CPT-1

A wholly different approach is proposed by the cross-protein transfer (CPT) modeling framework [59], a methodology that has demonstrated outstanding performance [14] but garnered relatively less attention. As devised through a feature selection process, CPT-1 utilizes a number of diverse features and scores. Firstly, it uses outputs from EVE and ESM-1v, complex models of sequence conservation and protein language, respectively. CPT-1 also directly utilizes MSAs from vertebrates and mammals at low depth, which likely has the most contribution to its increased performance, representing a local evolutionary timescale. Finally, it uses structure-based prediction scores from the ProteinMPNN [60] design tool and further uses structural information to constrain and the MSA-based scoring. Unlike most other top-performing VEPs, which strongly prioritize deep learning and big data approaches, CPT-1 combines its features for variant pathogenicity prediction through a simple linear regression model. What uniquely separates CPT-1 from most methods is that it was trained on MAVE data from only 5 proteins, while providing generalizable predictions for the proteome. In an independent benchmark, with its training data excluded, CPT-1 slightly outperformed AlphaMissense in a correlation task against functional assay values, demonstrating the potential power of creative training strategies and the importance of assay data.

### ESCOTT

ESCOTT [52] is an epistatic and structural model of mutational effects, derived from the GEMME architecture with modifications to also integrate several structure-derived features. It presents different scoring schemes depending on whether a variant is an un-structured region, buried in the protein core, or located at an interface. Respectively, the variants are simply evaluated using the evolutionary conservation from MSAs, or it is integrated together with terms reflecting atomic density surrounding the variant, or the physico-chemical properties of residues at an interface. Like GEMME, ESCOTT combines an independent and an epistatic term in its scoring scheme, estimating the number of changes required to accommodate a mutation over the entire sequence. Interestingly, although ESCOTT outperformed GEMME on a MAVE-based benchmark, GEMME slightly outperformed ESCOTT in the discrimination between pathogenic and putatively benign missense variants [14]; thus the benefit of including structural information over the already excellent GEMME is still not fully clear. The developers of the method also released an augmented version of the predictors, termed PRESCOTT, which utilizes population-specific allele frequency information, where available. This is intended to downweigh the predicted impact of common variants, but also introduces a risk of data circularity when tested for pathogenic *vs* benign variant discrimination, given that allele frequency is routinely used directly as evidence for clinical variant classification.

### Future avenues for variant effect predictor development are *complex*

Despite the apparent performance increase achieved through utilization of structural information, the few methodologies that include ablation analyses reveal that structure is not currently playing a massive role in their overall predictions [50,52,55,61,62]. Thus, it is likely that there is considerable room for improvement in how VEPs utilize protein structures. Additionally, while structure prediction methodologies have advanced considerably, they still fall short in dynamic structural regions, such as loops or intrinsically disordered termini, which may be functionally significant [63].

A clear limitation of the currently top-performing VEPs is that they do not explicitly take into account biomolecular complex structures or quaternary interactions when assessing the impacts of protein variants, which we know are important for almost all proteins *in vivo* [64]. While it has been demonstrated that sequence information is sufficient to identify intrachain contacts within proteins through coevolving residue positions, without training on properly paired MSAs of interolog sequences, it is much more difficult to capture the heteromeric interchain co-evolution between distinct interacting protein or nucleic acid sequences [65–68].

**Figure 3:**
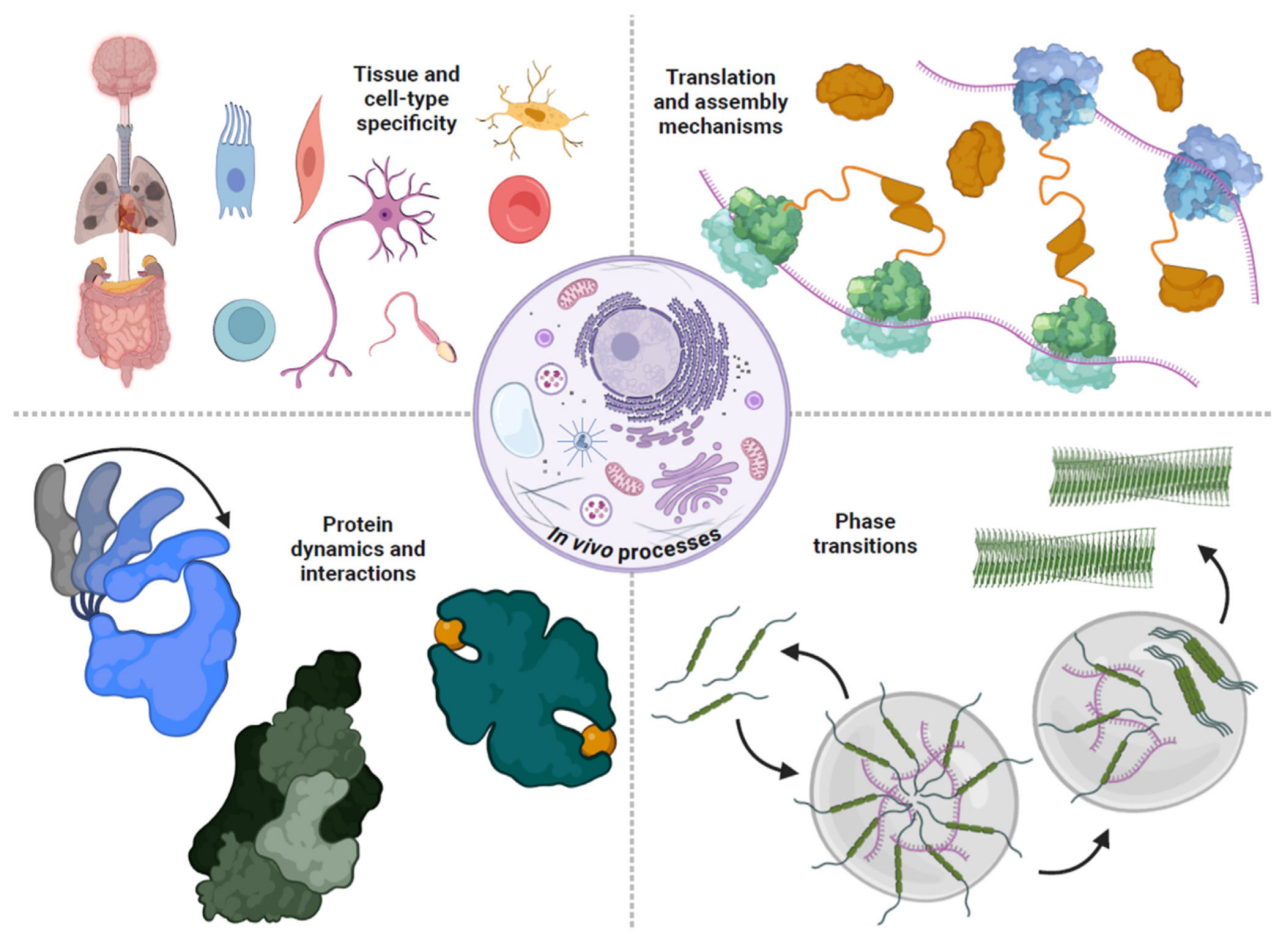
Various *in vivo* phenomena and processes involving proteins and their complexes need to be considered quantitatively to better understand the mechanisms and effects of variants in human cells. Tissue and cell-type specificity refers to the unique presence and concentrations of proteins, nucleic acids, and other interacting biomolecules, depending on the cell type, state and numerous environmental factors, which influence the possible intermolecular interactions *in vivo*. In cells, protein translation kinetics and assembly mechanisms can be affected by mutations, but are also modulated by phenomena like cotranslational assembly and domain arrangements, allowing the emergence of complex disease mechanisms. *In vivo*, missense mutations may manifest through altered protein conformational dynamics, affected allosteric networks, as well as mechanisms involving disruption of native and emergence of novel protein–protein or protein-ligand interactions. Finally, concentrations of interacting molecules may vary throughout the cellular environment, displaying different configurations in distinct phases, which would influence the effects of mutations in the different environments. Created in BioRender. Gerasimavicius, L. (2024) BioRender.com/i85u499.

Current methodologies are also not considering the various processes and phenomena that modulate and affect the outcomes of genetic mutations *in vivo* (Figure 3). Inside human cells, the concentrations of protein, nucleic acid, and small molecule binding partners will vary depending on the cell type, cell state, human developmental, and life stage and environmental factors. From the Human Cell Atlas project, which is in the era of assembling reference data objects for organs such as the brain and the lung, we know that there are on the order of thousands of cell types and cell states, each one with its own molecular fingerprint [69]. Each of these cell states will contain different concentrations of proteins, nucleic acids, and small molecules, which determine the intermolecular interactions of a protein. These interactions can occur both in a soluble aqueous phase or other microenvironments that incur state changes in a concentration-dependent manner, which predictors are now trying to capture systematically [70]. Furthermore, *in vivo*, the kinetics of protein translation and assembly can be influenced by both point mutations and domain arrangements [71]. Therefore, ideally, we would require quantitative structural analyses of complexes, containing proteins, nucleic acids, and ligands to not only reveal the effects of missense variants but also help interpret the likely molecular causes.

Training VEPs to accurately capture the functional variant effect landscape resulting from quaternary interactions would likely require an immense amount of diverse structural complex data, which is further complicated by the requirement that target proteins have a sufficient extent of observed benign or pathogenic variation. However, this effort could be driven by the very recent developments in protein structure prediction, with the release of AlphaFold 3 [72] and RoseTTAFold All-Atom (RFAA) [73], which both provide state-of-the-art generalized biomolecular frameworks for predicting protein–protein, protein-nucleic acid, and protein-ligand complex structures. AlphaFold 3 underwent another redesign, reducing the emphasis on MSA processing and shifting toward more information flow through the pair representation of proteins or nucleic acids in its new Pairformer framework. Importantly, the resource intensive structural module was abandoned in favor of a generative diffusion model, which means AlphaFold3 operates directly on raw atom coordinates, allowing prediction of multiple molecular types within the same complex. RFAA extends the previous three-track design to protein and nucleic acid inputs, and introduces graph representations for small-molecule ligands, offering the same prediction functionality as AF3, with the advantage of being open source.

While the current VEP methodologies using MSAs are able to capture most of the evolutionary signal pertaining to loss-of-function disease variants in singular subunits, they are not able to represent interactions and buffering that occur between variants in sequences that are not closely related, such as in heteromeric complexes. Depending on the alignment depth, such approaches also lack the capacity to separate unique contexts for related proteins, such as distinct spatiotemporal expression throughout tissues or within cells, leading to different interaction targets and unique effect landscapes. However, a recent study in *E. coli* has shown that an unsupervised genome-level language model was able to capture coevolution in protein complex interfaces, identifying functionally relevant interactions from genomic sequence stretches, essentially learning operon structure [74]. While this discovery was made in a prokaryotic context, the concept of training on genomic regions may be adapted to be of use in humans, providing an alternative approach to deriving complex structures.

Accurate prediction and downstream utilization of protein complex information is emerging as the new frontier in structural computational biology, which could open multiple new avenues for variant effect evaluation. Using advanced machine learning techniques, on top of providing improved loss-of-function variant prediction through identification of critical protein interfaces and their disruption, the effects of alternative molecular mechanism variants may be better captured by considering changes to complex-wide allosteric networks, disorder-to-order transitions of intrinsically disordered regions or shifts in preferred binding sites or partners. With the capacity to model any protein and nucleic acid complex offered by tools like AF3 or RFAA, we can more readily probe the effects of variation in transcription factors on DNA binding specificity [75], or even the inverse–elucidating the effects of noncoding DNA variation. The expanded capacity for protein-ligand complex prediction is also offering opportunities beyond disease variant evaluation, with the frameworks being applicable to pharmacogenomic variants and drug discovery. Moreover, while the focus of this review has been on VEPs that have excelled in scoring human variants, in principle these strategies could be applied to nonhuman sequences, offering insights for protein engineering, viral evolution, and other areas of research.

## Data Availability

No data was used for the research described in the article.
